# Entrepreneurial mindfulness and organizational resilience of Chinese SMEs during the COVID-19 pandemic: The role of entrepreneurial resilience

**DOI:** 10.3389/fpsyg.2022.992161

**Published:** 2022-10-06

**Authors:** Xuepeng Liu, Xiaohang Wu, Qing Wang, Zhenzhen Zhou

**Affiliations:** ^1^School of Management, Tianjin University of Commerce, Tianjin, China; ^2^Zhou Enlai School of Government, Nankai University, Tianjin, China; ^3^Department of Management Engineering, Qingdao University of Technology, Linyi, China

**Keywords:** entrepreneurial mindfulness, organizational resilience, entrepreneurial resilience, Chinese SMEs, COVID-19, entrepreneurs

## Abstract

Organizational resilience is vital to the survival and thriving of enterprises, especially during the COVID-19 pandemic. Although there has been an increasing interest in organizational resilience, the effects from the entrepreneur perspective receive scant attention. Based on upper echelons theory (UET) and personality psychology, we propose a model in which entrepreneurial mindfulness and entrepreneurial resilience could influence organizational resilience of SMEs. We empirically analyzed a sample of 180 entrepreneurs managing small- and medium-sized enterprises (SMEs) in China during the COVID-19 pandemic, using SmartPLS software. The research findings indicated that entrepreneurial mindfulness is positively associated with organizational resilience and such relationship is partially mediated by entrepreneurial resilience. These findings convey important theoretical implications in this field of research as well as practical implications for SMEs in China or other countries with similar nature.

## Introduction

As the forces of volatility, unpredictability, complexity and ambiguity (VUCA) intensify, organizations are presently operating in increasingly chaotic business environments (Liu et al., 2019). This is particularly true in the midst of the COVID-19 crisis which has created severe impact on business (Caligiuri et al., 2020). Organizations are now facing tremendous pressures for survival (Do et al., 2022). A crucial way for enterprises to survive and flourish is to become more resilient than ever before (Hillmann, 2021). Enterprises need to develop a resilience capacity to react to and capitalize on unexpected events which could potentially threaten the survival (Lengnick-Hall et al., 2011). Different from related constructs such as flexibility, agility or robustness, organizational resilience is an important success factor in dealing with unexpected threats and enabling firms to come out stronger than before. It helps enterprises to identify opportunities and challenges in adversity, get out of the crisis, and thus keep sustainable development (Ortiz-de-Mandojana and Bansal, 2016).

Since SMEs take a significant portion of the GDP and the livelihood conditions of people worldwide (Williams et al., 2013; Dahles and Susilowati, 2015; Sabatino, 2016; Alberti et al., 2018), fostering SMEs is deemed vital for the sake of tackling the socio-economic problems such as unemployment and poverty (Page and Söderbom, 2015). Although Demmer et al. (2011) suggested that factors influencing the resilience of large enterprises could also be applied to SMEs, it is revealed that SMEs can be significantly different from large enterprises in the way of operating and the degree of vulnerability (Sullivan-Taylor and Branicki, 2011). Organizational resilience may even be more critical for SMEs because of their vulnerability to various challenges caused by the limited size and resources (Branzei and Abdelnour, 2010; Tognazzo et al., 2016).

Since the outbreak of the COVID-19 pandemic started in China in early January 2020, the Chinese government has been adopting strict policies to curb the spread of the pandemic. In serious cases, resident activities were restricted to prevent community transmission, and companies were not allowed to resume operations. Till June of 2022, China has been suffering another serious outbreak of the pandemic in various provinces. For example, Shanghai, as the economic center of China and an important city of the world economy, had been put on complete lockdown for almost 100 days. During severe cases, more than 10,000 people were infected per day. Residents of almost the entire city were quarantined at home, and many companies were forced to stop working or working from home. As an important and rapidly growing country of the world economy, China deserves the research attention for the benefits of economic recovery and rejuvenation. Particularly, SMEs are the most dynamic enterprise group in China, accounting for about 60% of the GDP. However, they are highly vulnerable during global crises such as the COVID-19 pandemic, due to their limited resources and capabilities (Do and Shipton, 2019). In such turbulent times, organizational resilience can be vital for them to survive and thrive. Therefore, we choose the Chinese SMEs during the COVID-19 pandemic as our research context, which could offer opportunities to advance theories within the unique emerging market represented by China.

Scholars have extensively explored the factors affecting organizational resilience from both internal and external levels of organizations. Externally, social networks and cooperation are identified as important facilitators of organizational resilience. Internally, this dynamic capability can be influenced by factors of three different levels: the individual level (i.e., psychological capital, social capital (Chowdhury et al., 2019; Jia et al., 2020), entrepreneurial resilience (Manfield and Newey, 2017; Xiue and Mengying, 2020), employee resilience (Liang and Cao, 2021), etc.), the group level (i.e., team relationships (Faraj and Xiao, 2006), etc.), and the organizational level (i.e., organizational culture (Sawalha, 2015), organizational learning (Do et al., 2022), technology application (Zhang et al., 2021), etc.).

Specially for SMEs, Pal et al. (2014) identified three broad assets of key enablers for the organizational resilience in the Swedish textile and clothing industry: resourcefulness (material resources, financial resources, social resources, network resources, intangible resources), competitiveness (flexibility, redundancy of resources, robustness, networking), and learning and culture (leadership and top-management rapid decision-making, collectiveness and sense-making, employees well-being). Saad et al. (2021) reviewed the factors influencing SMEs’ resilience and categorized them into four key clusters: entrepreneurial characteristics (enterprise’s owner background, human capital, entrepreneurial orientation, and Social capital), firm internal resources (financial capital, size, business age, and types), external environment (socio-cultures, institutions, macro-economic conditions, location and infrastructures), and the interaction effects. In this paper, we combined the factors influencing SMEs’ organizational resilience into four categories: individual level, group level, organizational level, and business environment, which were summarized in Appendix. Corresponding references mentioned by the two studies are also shown in Appendix. Additionally, we also added to the summary with other new factors which were identified in recent years, including: (a) individual-level factors [life satisfaction and employee resilience (Prayag et al., 2020a,b), entrepreneurial resilience (Branicki et al., 2017), psychological capital and coping mechanisms (Prayag et al., 2020a,b), entrepreneurship (Herbane, 2019)]; (b) group level factors [collective mindfulness (Wang et al., 2021), collective rumination and group information processing (Knipfer and Kump, 2022)]; (c) organizational level factors [ambidexterity and strategic consistency (Iborra et al., 2020, 2022), dynamic capabilities (Ozanne et al., 2022), resource-based management initiative (Do et al., 2022), endowment, business ethic, altruism, loss aversion and herd behavior (Huang et al., 2022), digitalization, intellectual capital (Agostini and Nosella, 2022)]; and (d) business environmental factors [environmental dynamism (Do et al., 2022)].

Among the studies mentioned above, some explored the organizational resilience of SMEs within the background of the COVID-19 pandemic. For example, Ozanne et al. (2022) pointed that dynamic capabilities were critical in transform social capital into organizational resilience. Do et al. (2022) examined the mechanism between resource-based management initiatives (RBMI) and the organizational resilience of Vietnamese SMEs. Huang et al. (2022) found that altruism and business ethic were positively associated with organizational resilience of SMEs. Through a qualitative study of two SMEs, Agostini and Nosella (2022) revealed a tight connection between intellectual capital and organizational resilience.

Unlike large established enterprises in which the salience of entrepreneurs’ attributes are generally dissipated among wider governance and decision-making structures, SMEs are often highly influenced by the entrepreneurs (Branicki et al., 2017; Herbane, 2019). This perspective aligns with upper echelons theory (UET) that CEOs or top managers play a key role in shaping critical organizational outcomes (Hambrick and Mason, 1984; Hambrick, 2007; Carmeli et al., 2011). Also it corresponds to SME theory that acknowledges the centrality of the entrepreneur (Cornelius et al., 2006). During difficult times of the COVID-19 pandemic, it is imperative and rewarding to explore how entrepreneurs’ characters can influence the SMEs’ resilience.

It has been recognized that entrepreneurial resilience is critical for organizational resilience, especially for SMEs (Branicki et al., 2017; Duchek, 2020). Resilient entrepreneurs are more agile and flexible in times of adversity than non-resilient ones and have a higher propensity to take actions (Gorgievski and Stephan, 2016), which promotes the capabilities of coping and adaptation with regards to organizational resilience. However, among previous researches, the effect mechanism of entrepreneurial resilience on organizational resilience of SMEs has been still in the initial stage of research. Additionally, with regards to the antecedents of individual resilience, previous psychological researches have provided enough evidence that mindfulness plays an positive role for individuals in face of adversity and is positively related to individual resilience. Generally, mindfulness refers to a receptive attention to and an awareness of internal and external experiences as they occur (Brown et al., 2007). “Live in the present,” which is an ancient piece of advice, has been corresponded by the ever increasingly popularity of mindfulness in both academic and practical fields. Much evidence has shown the associations of mindfulness with individuals’ physical and psychological well-being (e.g., Keng et al., 2011; Hopwood and Schutte, 2017).

However, it still remains unclear about such association between mindfulness and resilience among the entrepreneurs group. Moreover, it is noteworthy that although deemed as an important positive psychological factor, entrepreneurial mindfulness has not been studied with regards to its effects on organizational resilience. Based on UET and personality psychology, this paper originally explores how the characters of entrepreneurial mindfulness and resilience influence organizational resilience of SMEs during the special context of the COVID-19 pandemic. In addition, we are also the first to explore the association between individual mindfulness and resilience among the entrepreneurs group.

Accordingly, this study examines the following three questions: (1) whether the characters of entrepreneurial mindfulness and resilience are positively associated with organizational resilience in the context of Chinese SMEs during the COVID-19 pandemic; (2) whether mindfulness and resilience at the individual level are positively correlated among the entrepreneurs group; and (3) whether entrepreneurial resilience mediates the relationship between entrepreneurial mindfulness and organizational resilience.

## Theoretical background and hypotheses

### Mindfulness and entrepreneurial mindfulness

#### Mindfulness

To define individual mindfulness is an arduous work because of its divergent definitions presented by different scholars. Conceptualized as a trait, mindfulness reflects individuals’ inclination to be mindful in daily life (Brown et al., 2007). Also, mindfulness can be regarded as a state in which an individual focuses his/her attention on present-moment events without non-intentional judgments. Distinct from similar present-moment focused states, such as absorption (Rothbard, 2001) and flow (Csikszentmihalyi and Csikzentmihaly, 1990), mindfulness at the individual level involves a wide breadth of attention from external events and phenomena to internal experiences (Dane, 2011). Empirical distinctions between mindfulness and flow were also given by Sheldon et al. (2015).

In the field of organizational psychology and behavior, definitions of individual mindfulness are more convergent. It is commonly conceptualized as a present-moment focused state of consciousness on ongoing physical, cognitive and psychological experiences in a nonjudgmental way. It has been generally recognized among organizational researches that individual mindfulness is positively related to a wide variety of employee performance factors including work engagement (Leroy et al., 2013), resistance to stress (Hulsheger et al., 2013), flexibility and creativity (Ie et al., 2012), and problem-solving skills (Olafsen, 2017), productivity, and job performance (Dane and Brummel, 2014).

#### Entrepreneurial mindfulness

Even though the effects of mindfulness on performance in organizational settings have been studied with samples of employees and leaders, mindfulness of entrepreneurs still remains largely under-explored. Only limited empirical researches demonstrated the positive relationships between entrepreneurial mindfulness and entrepreneurial outcomes. For example, Yener et al. (2018) demonstrated the positive and significant association between entrepreneurial mindfulness and entrepreneurship, with temperament playing as a mediating role. Tuan and Pham (2022) confirmed the positive relationships between mindfulness, perceived social support, and social entrepreneurial intention (SEI). Van Gelderen et al. (2019) discovered the positive effect of a high level of mindfulness on taking entrepreneurial actions was stronger for individuals who had prior entrepreneurial experience. Focusing on entrepreneurs’ subjective well-being, Yang et al. (2021) examined the moderating effect of mindfulness between entrepreneurial identity and work rumination.

During the COVID-19 pandemic, entrepreneurs in adversity are experiencing tremendous pressures. Generally, entrepreneurs of SMEs have a decisive influence on the organizational decision-making and performance. Specially, when the organization is in extremely crisis situations, an entrepreneur can play a vital role in rescuing and rejuvenating the enterprise, and even make it stronger than before. Therefore, it is absolutely imperative to conduct the research on organizational resilience from the side of entrepreneurs. From a cognitive point of view, mindfulness can lead to a great change in perspective, which in turn contributes to changes in behavior and positive outcomes (Shapiro et al., 2006). In this study, we are the first to examine the relationship between entrepreneurial mindfulness and organizational resilience. Possible results will provide us with effective initiatives in practice.

### Organizational resilience

Organizational resilience has been acknowledged as a critical source of sustainable competitive advantage for enterprises to survive in the severe VUCA era and to foster future success (Hamel and Valikangas, 2003; Sheffi, 2007). It has been conceptualized from various perspectives, such as proactive vs. reactive (Jia et al., 2020), planned vs. adaptive (Prayag et al., 2020a,b), external vs. internal (Marcucci et al., 2021), anticipation vs. reactive (Marcazzan et al., 2022). To date, most studies have extended the description of organizational resilience as offensive response (adaptation and/or anticipation), rather than just defensive response (resistance and/or recovery; Duchek, 2020; Ozanne et al., 2022). Still, little consensus has been reached regarding the conceptualization of organizational resilience. Generally, three categories of conceptualizations can be distinguished: (1) resilience as an outcome, (2) resilience as a process, and (3) resilience as an organizational capability. For example, Saad et al. (2021) conducted an extensive literature review SME resilience and created a definition as: “the SME’s adaptability to disruptions, growth (positive performance), and their ability to seize the business opportunity amid a challenging business environment.” Duchek (2020) defined organizational resilience as an organization’s ability to anticipate potential threats, to cope effectively with adverse events, and to adapt to changing conditions.

This study focuses on the combined perspective and considers resilience as a capability which can be developed and enhanced continuously in an organization, which echos the aim of our study to contribute to fostering SEMs’ capabilities to survive and succeed throughout different phases of the crisis. Based on the dynamic capability perspective as well as the integration of studies on high reliability organizations (HROs; Sutcliffe, 2011), crisis management and organizational learning, this paper identifies three dimensions of organizational resilience as anticipation, coping and adaptation, which is in line with that defined by Duchek (2020).

The first dimension of anticipation refers to determine how the environment is expected to change, and to bear in mind making decisions to avoid potential losses in highly ambiguous contexts (Madni and Jackson, 2009). It involves abilities of observing internal and external developments, identifying critical developments and potential threats, and preparing for unexpected events (Kendra and Wachtendorf, 2003; Somers, 2009; Burnard and Bhamra, 2011).

The second stage of coping refers to dealing with unexpected events after they have become manifest in quick response. It involves abilities of accepting a problem as well as developing and implementing solutions (Weick et al., 2005; Faraj and Xiao, 2006; Carmeli and Markman, 2011)。.

In line with organizational learning, the last stage of organizational resilience is adaptation, which refers to adjustments toward organizational advancement following crises. The underlying key capabilities of adaptation consist of reflection (or learning) and organizational change.

### Entrepreneurial mindfulness and organizational resilience

State mindfulness at the individual level includes two basic dimensions: (1) a sustained attention to and awareness of the present; and (2) a receptive, open, and nonjudgmental experiential processing (Davidson and Kaszniak, 2015; Good et al., 2016; Yu and Zellmer-Bruhn, 2018).

According to UET and SME theory, entrepreneurs’ characters play a key role in shaping organizational outcomes (Hambrick and Mason, 1984; Hambrick, 2007; Carmeli et al., 2011). Unlike large established enterprises in which the salience of entrepreneurs’ attributes are generally dissipated among wider governance and decision-making structures, SMEs are often highly influenced by the entrepreneurs (Branicki et al., 2017; Herbane, 2019). During difficult times of the COVID-19 pandemic, it is imperative and rewarding to explore how entrepreneurs’ characters can influence the SMEs’ resilience.

Prior to the unexpected crisis, entrepreneurial mindfulness enables entrepreneurs to allocate sustained attention and efficient awareness to the current enterprise both internally and externally. Based on the Conservation of Resource Theory (COR), this can lead to enhanced observation and quick identification of early crisis signals, which facilitates swift decision-making to avoid threatening situations or at least to minimize potential negative consequences in the future (McFarlane and Norris, 2006). Therefore, the anticipation stage of organizational resilience can be strengthened by the attention and awareness allocation of entrepreneurial mindfulness.

During the crisis, entrepreneurial mindfulness facilitates the information processing with features of receptiveness, openness and non-judgment. The nonreactive nature of perception reflected by receptiveness, the open-minded curiosity and compassionate intent revealed by openness can help entrepreneurs develop the ability of accepting the current unexpected crisis. In addition, open-minded curiosity can also enable entrepreneurs to focus on the development and brainstorm of coping strategies. Nonjudgmental information processing helps individuals response to the status quo as observed without immediate experiential judgment (Brown and Ryan, 2003; Good et al., 2016). Nonjudgmental process, together with receptiveness and openness, facilitates the emotional detachment of entrepreneurs, which helps to eliminate anxiety and stimulate positive affect. According to the Broaden-and-Build Theory, positive effects can help individuals to minimize their inner and outer conflicts, keep a positive attitude and cultivate a harmonious environment during the crisis (Fredrickson, 2004). So mindful entrepreneurs can focus on the current phenomena, facts, ideas, and resources. All of the above would contribute to the coping abilities of accepting the problem, as well as developing and implementing solutions for organizational resilience.

After the unexpected crisis, mindful entrepreneurs are generally able to concentrate constantly on the status quo of the entire organization system, as well as the values and goals. This helps to stabilize their awareness on recovering and prospering the enterprise, which would promote organizational reflection, learning and change for the transcendent and sustainable development. Moreover, an attitude of acceptance and an open mind can enable entrepreneurs to eliminate anxiety and keep optimistic about reflection and possible changes in the future. Thus, entrepreneurial mindfulness can also prompt organizational resilience after the unexpected crisis.

Therefore, we hypothesize that:

*H1*: Entrepreneurial mindfulness is positively associated with organizational resilience of SMEs.

### Entrepreneurial mindfulness and entrepreneurial resilience

Conceptualized as either a stable personality trait, a state-like exploitable capacity, a process, or an outcome (Connor and Davidson, 2003; Ong et al., 2006; Fisher et al., 2016; Hartmann et al., 2020), psychological resilience at the individual level refers to positive adaptation despite adversity (Richardson et al., 1990; Ahern et al., 2006). As the most encompassing perspective, the process conceptualization of psychological resilience involves the exposure to adversity and the response mechanism to adversities through cognition, affect and behavior (Ryff and Singer, 2003; Hoegl and Hartmann, 2021). Psychological resilience can endow entrepreneurs the abilities to bounce back in face of exceptionally stressful situations (Branicki et al., 2017; Santoro et al., 2020, 2021). Since the outbreak of the COVID-19 pandemic, enterprises have been afflicted chronically, which inevitably places tremendous pressures on entrepreneurs. Studies have demonstrated the positive link between entrepreneurial resilience and performance (Castro and Zermeño, 2020; Maritz et al., 2020; Nyikos et al., 2021; Purnomo et al., 2021; Mohammadifar et al., 2022).

Both intervention and correlation studies have suggested the positive relationship between mindfulness and resilience (Bajaj and Pande, 2016; McArthur et al., 2017; Pidgeon and Pickett, 2017; Roulston et al., 2018; Freligh and Debb, 2019; Vidic and Cherup, 2019; Reyes et al., 2020). The essence of mindfulness lies in the awareness of ongoing physical, cognitive and psychological experiences in a nonjudgmental way. From the perspective of cognitive theory, the decentralized mechanism of mindfulness promotes individuals’ awareness of their emotions, which can expand the space between stimulus and response (Shapiro et al., 2007; Verplanken and Fisher, 2014). Mindful individuals can perceive the status quo from a broader perspective, and thus acquire much cognitive flexibility toward the crisis (Thompson et al., 2011; Zou et al., 2020). They tend to maintain a calm, objective, open and receptive attitude, which could stimulate positive affects (Langer and Moldoveanu, 2000; Wallace and Shapiro, 2006; Tingaz, 2020). According to the Broaden-and-Build Theory, positive effects can help individuals to develop resilience in face of negative situations (Fredrickson, 2004; Johnson et al., 2021). Studies from neuroscience have also shown that mindfulness can reduce the amygdala response in the brain which triggers negative emotions, thus making individuals more happy, peaceful and stable (Davidson and Begley, 2012; Barnhofer et al., 2021; Medina et al., 2022).

Mindful individuals tend to keep a sustained attention to the present situation of the organization internally and externally, which enables them to preserve resources by neglecting distractions and concentrate on coping with the present situation (Wimmer et al., 2016; Dubey et al., 2020). From the perspective of COR, individuals have the tendency to preserve, protect and acquire resources (Montani et al., 2018; Nemțeanu and Dabija, 2021; Nemțeanu et al., 2022). The positive affects acquired by entrepreneurial mindfulness are psychological resources for entrepreneurs to bounce back from adversity. In addition, From the literature above, we assume that mindful entrepreneurs tend to be more resilient during the crisis of the COVID-19 pandemic.

Therefore, we propose the following hypothesis:

*H2*: Entrepreneurial mindfulness is positively associated with entrepreneurial resilience.

### Entrepreneurial resilience and organizational resilience

According to UET, managerial characteristics and beliefs of certain key individuals can impact the firm’s strategic choices (Hambrick and Mason, 1984; Hambrick, 2007). Compared with large established firms, the influence of core individuals in SMEs is likely to have greater impact on organizational outcomes, because of the relatively smaller sizes and less hierarchy. This also aligns with SME theory which acknowledges the centrality of the entrepreneur (Cornelius et al., 2006). The founding entrepreneur is at the core of the SME, and is the key determinant of the firm’s strategic decisions (Huang et al., 2012; Bjornali et al., 2016; Ahn et al., 2017).

In times of adversity, resilient entrepreneurs are more agile and flexible than non-resilient individuals and have a higher propensity to take actions (Gorgievski and Stephan, 2016), which promotes the capabilities of coping and adaptation with regards to organizational resilience. In addition, as decision makers of SMEs, resilient entrepreneurs can better spread their resilient culture among employees through leadership and knowledge sharing (Suppiah and Sandhu, 2011; Cassia et al., 2014). As a result, the collective resilience of all individual members in the organization could lead to a positive response to the crisis with collective concentration on coping and adaptation, which leads to the enhancement of organizational resilience.

Therefore, even though entrepreneurial resilience is indeed an individual-level construct, it is viewed as highly influential on organizational resilience, especially for SMEs (Duchek, 2020). We hypothesize that:

*H3*: Entrepreneurial resilience is positively associated with organizational resilience of SMEs.

### Mediating effect of entrepreneurial resilience between entrepreneurial mindfulness and organizational resilience

We have thus far argued that entrepreneurial mindfulness can enable SMEs to build resilience in response to crises effectively. However, entrepreneurial mindfulness may be necessary but insufficient in developing organizational resilience. As in sections Entrepreneurial mindfulness and entrepreneurial resilience and Entrepreneurial resilience and organizational resilience, two additional arguments for SMEs have been established. Firstly, entrepreneurial mindfulness may be positively associated with entrepreneurial resilience. And secondly, entrepreneurial resilience may be positively correlated with organizational resilience. Combining the two sets of arguments, we propose the mediating role of entrepreneurial resilience. In other words, entrepreneurs need to transform their emotion and attention resources accumulated from individual mindfulness into resilient behaviors which can effectively foster organizational resilience.

Although, according the UET, entrepreneurs’ psychological or behavioral characters can create critical influences on organizational outcomes (Chen et al., 2020; Liu et al., 2021) even in the SME context (Barrett et al., 2021; Bennat and Sternberg, 2022), the mediating effect of entrepreneurial resilience between entrepreneurial mindfulness and organizational resilience have never been explored, especially in the context of the COVID-19 pandemic. Thus, we focus on the indirect relationship between entrepreneurial mindfulness and organizational resilience through entrepreneurial resilience, and propose the fourth hypothesis as below.

*H4*: Entrepreneurial resilience mediates the relationship between entrepreneurial mindfulness and organizational resilience of SMEs.

## Methodology

### Data collection and sample

In this study, we conducted an online survey to collect data among SMEs in China. To ensure a valid data base, we adopted both snowball sampling and stratified sampling. Firstly, by making adequate use of social networks (friends, alumni, co-workers), we employed snowball sampling in order to get access to respondents of interest to form our sample as much as possible.

To deal with potential problems associated with single-informant bias and common method bias, we collected data in two phases. At Time 1, a questionnaire link was sent *via* email or WeChat to the entrepreneurs with a cover letter introducing the ethical issues and objectives of the study, as well as the confidentiality and usage of the data gathered. The questionnaire at Time 1 was for entrepreneurs to provide information with regards to the variables of entrepreneurial mindfulness and resilience, as well as the control variables. Respondents were asked if he or she was the founder or decision maker while managing the enterprise. If not, the response would be discarded.

In addition, we also encouraged the recipients to share with other entrepreneurs the link of the questionnaire. This helped reduce possible desirability bias because of the completely confidential promise provided in the cover letter of the questionnaire.

As a result, 356 responses were collected. On the return of surveys, we used stratified sampling with firm size to filter participants (Mathew et al., 2013). This is to ensure precise estimates for the target population of SMEs in China. Response from large-sized companies (over 500 employees) were excluded. Then we focus on the remaining 228 participants.

After 3 months, at Time 2, we finally collected the information of organizational resilience from the entrepreneurs. The final complete sample includes 180 participants (a 50.5% response rate), which is described in Table 1.

**Table 1:** Sample descriptions (*N* = 180).

Descriptive characteristics		Frequency	Percentage %
Entrepreneurs gender	1 = male	102	56.67%
	0 = female	78	43.33%
Entrepreneurs age	1. Less than 25 years	12	6.67%
	2. 25–34 years	27	15.00%
	3. 35–44 years	104	57.78%
	4. 45–54 years	19	10.56%
	5. 55 years and older	18	10.00%
Firm age	1. Less than 1 years	1	0.56%
	2. 1–2 years	7	3.89%
	3. 3–4 years	16	8.89%
	4. 5–8 years	65	36.11%
	5. More than 8 years	91	50.56%
Firm size	1. Less than 10 employees	10	5.56%
	2. 11–50 employees	46	25.56%
	3. 51–100 employees	66	36.67%
	4. 101–200 employees	35	19.44%
	5. 201–500 employees	23	12.78%
Industry	1 = manufacturing	38	21.11%
	0 = others	142	78.89%

### Measures

The instruments of entrepreneurial mindfulness and entrepreneurial resilience, which were adopted in this paper, were originally developed in English. They were subsequently translated into Chinese in accordance with the proposed back-translation method (Brislin, 1970). To measure organizational resilience, we used the instrument developed specially for enterprises in the Chinese scenario. All items of the constructs in our model were quantified on a five-point Likert-type scale, which are shown in Table 1. The measurements of the constructs are described in the following:

#### Entrepreneurial mindfulness

The variable was measured using the eight-items “Freiburg Mindfulness Inventory” developed by Kohls et al. (2009). It is deemed as a second-order construct measured by two dimensions: *acceptance* and *presence*. Each dimension is assessed using four items.

#### Entrepreneurial resilience

We assessed this variable with the brief resilience coping scale developed by Sinclair and Wallston (2004). It describes the ability of entrepreneurs to cope with crises and adversities (Folkman and Moskowitz, 2000; Tedeschi and Calhoun, 2004).

#### Organizational resilience

The organizational resilience scale adopted in this paper was developed by Zhang and Teng (2021). Based on semi-structured interviews and previous organizational resilience measurements (Home III and Orr, 1997; Edmondson, 2002; Kendra and Wachtendorf, 2003; Masys, 2005; Lee et al., 2013; Duchek, 2020), the scale was developed specially for enterprises in the Chinese scenario. It is a second-order construct with 15 items, measured by three dimensions: *anticipation* (five items); *adaptability* (six items); and *situation awareness* (four items). Although the three dimensions do not one-to-one correspond to the three stages (anticipation, coping, and adaptation) of organizational resilience mentioned in section Theoretical background and hypotheses, the total items can reflect the mentioned capabilities, such as observation, identification, preparation, accepting, developing and implementing solutions, reflection or learning and organizational change.

#### Control variables

Finally, we controlled for entrepreneurs age (Age) and gender (Gender), which could affect entrepreneurial resilience (Ayala and Manzano, 2014). Additionally, at the firm level, firm age, size and industry were used as control variables for organizational resilience, which is in line with what Shirokova et al. (2016) suggested for SMEs. As shown in Table 1, we included a dummy variable “industry,” with 1 meaning manufacturing services and 0 meaning other industries, as well as a dummy variable “entrepreneurs gender,” with 1 meaning male services and 0 meaning female.

### Empirical method

Previous studies have demonstrated that partial least squares (PLS) is an established and robust method for studies in business (Carrión et al., 2016) and strategic management research (Hair et al., 2014). PLS is deemed as the method of choice whenever the research is exploratory or at the early stages of theory development and whenever the sample size is small (Reinartz et al., 2009). Given the relatively small sample size of 180 and that the proposed model has not been tested before, PLS is very suitable for our present study.

We utilized SmartPLS 3.0 to conduct both the measurement assessment and structural model test, which were suggested as the proper execution of PLS statistical analysis (Reinartz et al., 2009). First, the measurement models were validated, and then the structural model was tested by applying non-parametric bootstrapping with 5,000 replications and mean replacement of missing values (Hair et al., 2012).

### Common method bias

Since all variables were collected by the same respondent, attention was paid to the issue of common method bias (CMB). We addressed the issue ex-ante and ex-post to the data collection phase. To minimize CMB ex-ante, we took the following measures proposed by Podsakoff et al. (2003). Firstly, we adopted established measurement scales, assured respondent anonymity, and set the questions in a counterbalancing order. Secondly, the dependent variables and the independent variables were collected in different time periods.

Following the data collection phase, we firstly conducted the Harman’s single factor test (Podsakoff and Organ, 1986), only to find that the single factor just explained 49.33% of the variance, which indicated that the common method should not be an issue (Podsakoff et al., 2003). Furthermore, we adopted the unmeasured method factor approach for PLS-SEM to check for CMB (Liang et al., 2007). As shown in Table 2, the average substantively explained variance of all indicators is 0.645, and average method-based variance is 0.017, which yields a ratio of 37.9:1. This confirms that CMB is unlikely to be a serious concern for our study. Last but not the least, as all variance inflation factors between the first-order constructs were below 5, we concluded that multicollinearity did not indicate common method bias (Kock, 2015). Therefore, common method bias was not a serious issue in our study.

**Table 2:** Common method factor analysis for CMB.

Latent constructs	Indicators	Substantive factor loading (R1)	R1^2^	Method factor loading (R2)	R2^2^
EM	EM1	0.562^***^	0.316	0.143	0.020
	EM2	0.733^***^	0.537	−0.139	0.019
	EM3	0.727^***^	0.529	0.116	0.013
	EM4	0.688^***^	0.473	0.120	0.014
	EM5	0.808^***^	0.653	−0.011	0.000
	EM6	0.931^***^	0.867	−0.182^*^	0.033
	EM7	0.863^***^	0.745	−0.101	0.010
	EM8	0.724^***^	0.524	0.031	0.001
ER	ER6	0.887^***^	0.787	0.022	0.000
	ER7	0.926^***^	0.857	−0.016	0.000
	ER8	0.874^***^	0.764	0.031	0.001
	ER9	0.931^***^	0.867	−0.037	0.001
OR	OR1	0.536^***^	0.287	0.159	0.025
	OR2	0.827^***^	0.684	−0.042	0.002
	OR3	0.460^***^	0.212	0.341^*^	0.116
	OR4	0.883^***^	0.780	−0.106	0.011
	OR5	0.824^***^	0.679	−0.018	0.000
	OR6	0.724^***^	0.524	0.079	0.006
	OR7	0.970^***^	0.941	−0.177	0.031
	OR8	0.751^***^	0.564	0.060	0.004
	OR9	0.741^***^	0.549	0.059	0.003
	OR10	1.062^***^	1.128	−0.325	0.106
	OR11	0.829^***^	0.687	−0.030	0.001
	OR12	0.627^***^	0.393	0.134	0.018
	OR13	0.840^***^	0.706	−0.060	0.004
	OR14	0.735^***^	0.540	0.023	0.001
	OR15	0.914^***^	0.835	−0.086	0.007
AVE		0.792	0.645	−0.001	0.017

EM, Entrepreneurial Mindfulness; ER, Entrepreneurial Resilience; OR, Organizational Resilience; AVE, Average.

*** *p* < 0.001;

* *p* < 0.05.

## Results

### Evaluation of measurement model

To ensure the quality of our measures, all psychometric properties of the reflective measured constructs were assessed in SmartPLS according to commonly agreed indicators for reliability and validity (Hair et al., 2011; Table 3). As shown in Table 3, the standardized factor loadings of all items in our measurement model ranged from 0.763 to 0.919, which ensured an adequate indicator reliability. The results in Table 3 also illustrated the values of composite reliability ranging from 0.885 to 0.956 and Cronbach’s ɑ ranging from 0.741 to 0.942, which also supported the internal consistency of our constructs. By computing the average variance explained per factor, we found the convergent validity was substantiated since these values exceeded 0.5. In addition, all AVE values exceeded the highest squared inter-construct correlations, which confirmed the discriminant validity of our constructs (Table 4; Fornell and Larcker, 1981). Furthermore, the heterotrait–monotrait ratio of correlations (HTMT) was below the threshold of 0.85 (Table 5), which also indicated that the discriminant validity was established (Henseler et al., 2015).

**Table 3:** Quality criteria of reflective first-order-constructs.

Construct	Measurement item	Item loadings	Cronbach’s alpha	CR	AVE
Entrepreneurial mindfulness			0.741	0.885	0.794
Acceptance	I am able to appreciate myself.	0.767	0.844	0.896	0.683
	In difficult situations, I can pause without immediately reacting.	0.763			
	l am friendly to myself when things go wrong.	0.907			
	I experience moments of inner peace and ease, even when things get hectic and stressful.	0.862			
Presence	I am open to the experience of the present moment.	0.858	0.883	0.919	0.741
	When I notice an absence of mind, I gently return to the experience of the here and now.	0.857			
	I pay attention to what is behind my actions.	0.885			
	I feel connected to my experience in the here-and-now.	0.842			
Entrepreneurial resilience	I actively look for ways to replace the losses I encounter in life	0.905	0.926	0.947	0.818
	I believe that I can grow in positive ways by dealing with difficult situations	0.913			
	I look for creative ways to alter difficult situations	0.900			
	Regardless of what happens to me, I believe I can control my reaction to it	0.900			
Organizational Resilience			0.865	0.917	0.787
Adaptability	In the event of a crisis, our organization shows a strong attitude of acceptance.	0.814	0.931	0.946	0.745
	Our organization can successfully learn lessons from past or current projects, and ensure that these lessons be implemented into future projects.	0.893			
	Our organization can quickly shift from usual patterns to crisis-response modes.	0.885			
	Our organization can quickly get access to the resources needed to deal with accidents during a crisis.	0.838			
	In the event of a crisis, our organization can establish a collective coordination mechanism to ensure the organization in a state of system-wide response.	0.882			
	When a crisis occurs, our organization has the ability to adapt itself to circumstances and solve problems creatively.	0.863			
Anticipation	Our organization has been ready to deal with emergencies and take advantage of unforeseen opportunities.	0.908	0.942	0.956	0.812
	Our organization proactively monitors the present status of industry, which facilitates early warnings of emerging problems.	0.916			
	Our employees can usually make the time from their routine roles to practice how to deal with emergencies.	0.904			
	Our employees know how soon the organization will be affected by unexpected and potential negative events.	0.884			
	Our organization can not only observe and identify actual changes and upcoming crises, but also focus on potential future developments.	0.894			
Situation awareness	During a crisis, our employees communicate frequently enough to catch on what the organization is going through.	0.859	0.909	0.936	0.786
	Our organization can deploy personnel to fill key vacancies at any time.	0.890			
	Our organization realizes that the success or failure of each department within the organization is closely linked with each other.	0.878			
	Our organization understands the minimum level of resources required to operate successfully.	0.919			

**Table 4:** Descriptive statistics and construct correlations.

Construct			Mean	Std. Dev.	1	2	3	4	5	6
Entrepreneurial mindfulness	1	Acceptance	3.874	0.651	0.827					
	2	Presence	4.022	0.602	0.600	0.861				
Organizational resilience	3	Adaptation	4.307	0.658	0.510	0.456	0.863			
	4	Anticipation	4.027	0.790	0.531	0.446	0.642	0.901		
	5	Situation awareness	4.031	0.775	0.493	0.470	0.707	0.702	0.887	
Mediator	6	Entrepreneurial resilience	4.160	0.623	0.574	0.577	0.625	0.517	0.569	0.905

Numbers on the main diagonal show the square-root of the AVE.

**Table 5:** The heterotrait-monotrait ratio of correlations.

	Acceptance	Presence	Adaptation	Anticipation	Situation Awareness	Entrepreneurial resilience
Acceptance						
Presence	0.689					
Adaptation	0.571	0.502				
Anticipation	0.590	0.488	0.682			
Situation awareness	0.559	0.526	0.766	0.757		
Entrepreneurial resilience	0.643	0.638	0.673	0.553	0.619	

In order to measure the hierarchical second-order constructs, we used the type II reflective-formative approach based on the repeated indicator approach (Becker et al., 2012). As shown in Table 6, the path weights of the first-order reflective constructs to the second order formative constructs were all significant. The multicollinearity among the first-order constructs was tested using the variance inflation factors, all of which were below the threshold of 5 (Table 6). Therefore, multicollinearity was not an issue for our constructs (Hair et al., 2011).

**Table 6:** Evaluation of the inner formative measurement model.

Construct/item	Path weight	*t*-value	VIF
**Entrepreneurial mindfulness**			
Acceptance	0.562^***^	46.261	1.529
Presence	0.560^***^	44.143	1.529
**Organizational resilience**			
Adaptation	0.377^***^	39.995	2.157
Anticipation	0.368^***^	35.490	2.134
Situation awareness	0.382^***^	51.828	2.503

*** *p* < 0.001.

### Evaluation of structural model

**Figure 1:**
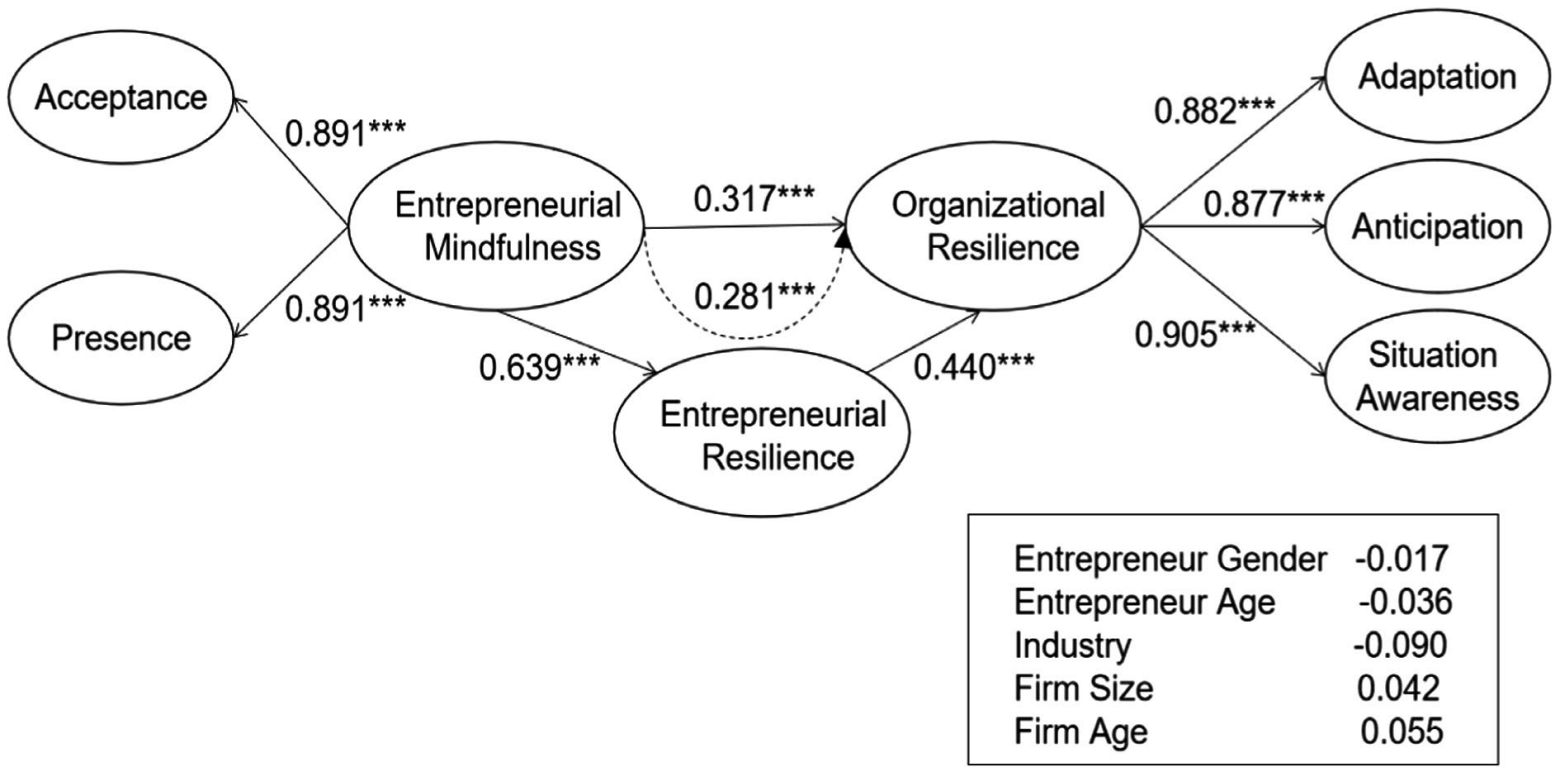
Full model analysis results. ****p < 0.001.

First, we examined the inner VIF values of the model, all of which were less than the threshold of 5, suggesting that multicollinearity is not an issue (Hair et al., 2017). Second, we assessed the relationships we hypothesized in this paper using path coefficients. As shown in Figure 1, entrepreneurial mindfulness had a significant impact on entrepreneurial resilience (*β* = 0.639 *t* = 11.624, *p <* 0.001) and on organizational resilience (*β* = 0.317, *t* = 4.499, *p <* 0.001). At the same time, entrepreneurial resilience also had a statistically significant impact on organizational resilience (*β* = 0.440, *t* = 5.948, *p <* 0.001). Consequently, the results supported our hypotheses 1, 2, and 3 (see Table 7). The control variables produced no significant effects (*p* > 0.1).

**Table 7:** Hypothesis testing.

Paths	*β*	*t-*value	*p-*value	2.5%	97.5%	$f^{2}$	Hypothesis
**Control variables**							
Entrepreneurs age → ER	−0.036	0.665	0.506	−0.142	0.067	0.002	
Entrepreneurs gender → ER	−0.017	0.137	0.891	−0.264	0.231	0.000	
Firm age → OR	0.055	1.013	0.311	−0.055	0.162	0.005	
Firm size → OR	0.042	0.706	0.480	−0.074	0.159	0.003	
Industry → OR	−0.090	0.583	0.560	−0.410	0.200	0.002	
**Direct effect**							
EM → OR	0.316^***^	4.265	0.000	0.177	0.469	0.113	H1 supported
EM → ER	0.621^***^	9.431	0.000	0.488	0.742	0.683	H2 supported
ER → OR	0.483^***^	5.915	0.000	0.309	0.631	0.212	H3 supported
**Mediation analysis**							
Step 1 (Indirect effect): EM → ER → OR	0.300^***^	5.648	0.000	0.194	0.401		Partial mediation—H4 partially supported
Step 2 (Direct effect): EM → OR	0.483^***^	5.915	0.000	0.177	0.469		

EM, Entrepreneurial Mindfulness; ER, Entrepreneurial Resilience; OR, Organizational Resilience.

*** *p* < 0.001.

Third, we assessed the predictive accuracy and effect sizes of our model and the results are shown in Table 8. The values of R^2^ ranged from 40.9% to 48.1%, suggesting that the predictive power of our model is between substantial and medium. We also assessed the predictive relevance of our model with the *Q^2^* statistics calculated through an omission distance of 7. As a result, the values of all exogenous constructs were above zero, suggesting significant predictive relevance. Additionally, we examined the effect size *f^2^* to assess the exogenous variable’s contribution to the *R^2^* value of the endogenous variable (Hair et al., 2017). Table 7 provides *f^2^* values of supported hypotheses H1, H2, and H3 with medium and large effects.

**Table 8:** Predictive relevance and effect size.

Endogenous variables	$R^{2}$	$Q^{2}$ (=1 − SSE/SSO)	Exogenous variables	Effect size $f^{2}$
Entrepreneurial resilience	0.409	0.323	Entrepreneurial mindfulness	0.683
Organizational resilience	0.481	0.365	Entrepreneurial mindfulness	0.113
			Entrepreneurial resilience	0.212

### Mediating effects

We conducted the assessment of mediation effects in our model by following the two-step procedure proposed by Nitzl et al. (2016). Firstly, the significance of the indirect effect was examined, only to find the indirect effect of entrepreneurial mindfulness on organizational resilience through entrepreneurial resilience is significant (*β* = 0.300, *t* = 5.648, *p <* 0.001). Then we examined the direct effect to determine the mediation type. As shown in Table 7, the direct effect of entrepreneurial mindfulness on organizational resilience is significant (*β* = 0.483, *t* = 5.915, *p <* 0.001), which suggested the partial mediation effect of entrepreneurial resilience between entrepreneurial mindfulness and organizational resilience. In other words, H4 is partial supported.

We adopted the variance-accounted-for (VAF) value to evaluate the mediation effect strength (Nitzl et al., 2016). Table 7 shows that the VAF value for entrepreneurial resilience is 38.31%, which demonstrates the ratio of indirect effect to total effect.

### Robustness checks

We conducted two analysis on potential non-linearity effect and endogeneity (Hult et al., 2018; Sarstedt et al., 2020; Ozanne et al., 2022) respectively to check the robustness of our model. To assess the nonlinear effects, we first conducted Ramsey’s (1969) RESET test in RStudio, using latent variable scores extracted from the original model’s PLS-SEM algorithm. As a result, neither the partial regression of OR on EM and ER (*F* = 1.2725, *p* = 0.2608) and that of ER on EM (*F* = 1.6482, *p* = 0.2009) are shown significant (see Table 9). Next, we examined the quadratic effects of EM and ER on OR and EM on ER. All quadratic effects are insignificant, suggesting the linear effects of our model are robust.

**Table 9:** Assessment of nonlinear effects.

Nonlinear relationship	Coefficient	*p-* value	$f^{2}$	Ramsey’s RESET
EM^*^EM → OR	−0.066	0.137	0.010	*F* = 1.2725, *p* = 0.2608
ER^*^ER → OR	0.056	0.228	0.008	
EM^*^EM → ER	0.056	0.241	0.009	*F* = 1.6482, *p* = 0.2009

EM, Entrepreneurial Mindfulness; OR, Organizational Resilience; ER, Entrepreneurial Resilience.

* *p* < 0.05.

To test the potential endogeneity, we followed the systematic procedure proposed by Hult et al. (2018). By using the latent variable scores of the original model estimation as input, we applied Park and Gupta’s (2012) Gaussian copula approach in RStudio. Firstly, *via* the Kolmogorov–Smirnov test with Lilliefors correction, all independent latent variables (i.e., ER and EM) were verified to be non-normally distributed, which is a required condition. Then three regression models were established in RStudio, which includes all possible combinations of Gaussian copulas. The results in Table 10 show that cEM and cER are significant in model 1 and model 2 (*p* < 0.05), suggesting a potential endogeneity problem. Note that we have also checked the combination of Gaussian copulas (cEM and cER) included in model 3, only to find neither of them are significant (Table 10). However, when dealing with endogeneity with the Gaussian Copula approach proposed by (Hult et al., 2018), we found the significance of EM and ER did not change from those in the original value, which supports the robustness of our model results.

**Table 10:** Results of the Gaussian Copula approach.

Variable	Original Model		Gaussian copula Model 1 (endogenous variable: EM)		Gaussian copula Model 2 (endogenous variable: ER)		Gaussian copula Model 3 (endogenous variable: EM, ER)	
	Value	*p-* value	Value	*p-* value	Value	*p-* value	Value	*p-* value
EM	0.38768	<0.001	0.54405	<0.001	0.458877	<0.001	0.510152	<0.001
ER	0.44730	<0.001	0.48237	<0.001	0.566100	<0.001	0.516459	<0.001
cEM			−0.03783	0.0190812			−0.024306	0.4676413
cER					−0.029315	0.03906553	−0.013225	0.6502893

EM, Entrepreneurial Mindfulness; OR, Organizational Resilience; ER, Entrepreneurial Resilience.

### *Post-hoc* analysis

In addition to the first-order construct of entrepreneurial mindfulness, we assessed the influence of the second-order constructs of *acceptance* and *presence* on organizational resilience through the entrepreneurial resilience as a mediator. As a result, little difference was found between the results of second-order and first-order constructs.

In Table 11, we found that *acceptance* had a significant direct influence on organizational resilience (*β* = 0.271 *t* = 3.626, *p* < 0.001), and the indirect impact was also significant (*β* = 0.154, *t* = 3.853, *p* < 0.001). It demonstrated that entrepreneurial resilience partially mediated the relationship between *acceptance* and organizational resilience.

**Table 11:** First-order constructs and organizational resilience.

Paths	*β*	*t*-value	*p-* value
**Control variables effects**			
Entrepreneurs age → Entrepreneurial resilience	−0.037	0.709	0.478
Entrepreneurs gender → Entrepreneurial resilience	−0.020	0.158	0.874
Industry → Organizational resilience	−0.057	0.364	0.716
Firm size → Organizational resilience	0.038	0.647	0.518
Firm age → Organizational resilience	0.053	0.955	0.339
**Direct effects**			
Acceptance → Entrepreneurial resilience	0.357^***^	4.771	0.000
Acceptance → Organizational resilience	0.271^***^	3.626	0.000
Presence → Entrepreneurial resilience	0.366^***^	4.765	0.000
Presence → Organizational resilience	0.101	1.297	0.195
Entrepreneurial resilience → Organizational resilience	0.430^***^	5.661	0.000
**Indirect effects**			
Acceptance → Organizational resilience	0.154^***^	3.853	0.000
Presence → Organizational resilience	0.158^***^	3.867	0.000
**Total effects**			
Acceptance → Organizational resilience	0.424^***^	5.541	0.000
Presence → Organizational resilience	0.259^**^	3.104	0.002

*** *p* < 0.001;

** *p* < 0.01.

With regards to *presence*, its indirect effect on organizational resilience was significant (*β* = 0.158, *t* = 3.867, *p* < 0.001), while the direct impact was not significant (*β* = 0.101, *t* = 1.297, *p* = 0.195). It revealed that the relationship between *presence* and organizational resilience was fully mediated by entrepreneurial resilience.

We further found that *acceptance* and *presence* had a significant influence on entrepreneurial resilience (*β* = 0.357, *t* = 4.771, *p* < 0.001 and *β* = 0.366, *t* = 4.765, *p* < 0.001) respectively.

Additionally, the total effects of *acceptance* and *presence* on organizational resilience were significant (*β* = 0.424, *t* = 5.541, *p* < 0.001 and *β* = 0.259, *t* = 3.104, *p =* 0.002 < 0.01) respectively. All controlled factors showed no significant impact in the model.

## Discussion and implication

### Discussion of findings

We examined how entrepreneurial mindfulness influences organizational resilience through the mediating mechanism of entrepreneurial resilience within a unique SME context of China during the COVID-19 crisis. The empirical results supported our original theoretical predictions that entrepreneurial mindfulness is positively associated with entrepreneurial resilience and organizational resilience, respectively. In other words, within the Chinese SME context, the entrepreneurial mindfulness contributes to the resilience at the entrepreneurial level as well as the organizational level. Our findings also supported that entrepreneurial resilience is positively associated with organizational resilience, suggesting that entrepreneurial resilience spurs the resilience at the organizational level for Chinese SMEs during the COVID-19 pandemic. Moreover, the results demonstrated partial support for our hypothesis about the mediating effect of entrepreneurial resilience between entrepreneurial mindfulness and organizational resilience. Our model was demonstrated to possess enough predictive power and robustness. In addition, the *post-hoc* analysis results did not invalidate our main results.

### Theoretical implications

This study contributes to theory in different ways.

First, building on UET and personality psychology, this study enriches the body of researches on organizational resilience by considering the influence of entrepreneurial mindfulness. Although there has been an increasing interest in organizational resilience, the effects from the entrepreneur perspective have received less attention. Compared with large established firms, SMEs tend to be more influenced by entrepreneurs, especially in extremely crisis situations such as the COVID-19 pandemic. There have been limited researches explored the organizational resilience of SMEs from the perspective of entrepreneurial resilience and entrepreneurship. However, it is noteworthy that although deemed as an important positive psychological factor, entrepreneurial mindfulness has never been studied as an antecedent of organizational resilience. Our results shed light on the organizational resilience literature by confirming entrepreneurial mindfulness as an important antecedent of organizational resilience for SMEs during a crisis. Therefore, it can also enrich the entrepreneurship theory provide from a new perspective.

Second, as revealed by our gap analysis, our study adds to the body of research on individual mindfulness among the population of entrepreneurs, which has received scant attention in literature. Our results supported that entrepreneurial mindfulness is positively related to entrepreneurial resilience. As demonstrated by our study, the positive link between entrepreneurial mindfulness and entrepreneurial resilience is in consonance with the results of previous studies that mindfulness and resilience are positively correlated at the individual level among populations of clinic, students, employees, etc. Specifically, mindfulness enables entrepreneurs to allocate sustained attention and efficient awareness to the current situation with a receptive, open and nonjudgmental state of mind, which endows entrepreneurs with enough internal resources and positive affects to deal with the crisis across periods of anticipation, coping and adaptation.

Third, although there has been research revealing the positive link between entrepreneurial resilience and organizational resilience (Manfield and Newey, 2017; Xiue and Mengying, 2020), never has its joint influence with entrepreneurial mindfulness on organizational resilience been explored. The findings support that entrepreneurial resilience is positively associated with organizational resilience. It is in consonance with previous studies which demonstrated that entrepreneurial resilience facilitates organizational resilience especially in small firms (Duchek, 2020). Resilient entrepreneurs tend to be agile and flexible in times of adversity and have a high propensity to take actions, which can be spread as a resilient culture among the whole organization because of the relatively small sizes. As a result, the collective resilience of all individual members in the organization can lead to positive responses to the crisis with collective concentration on coping and adaptation, which enhances organizational resilience. Imperatively, our results show that entrepreneurial resilience partially mediates the relationship between entrepreneurial mindfulness and organizational resilience for SMEs during the COVID-19 pandemic. Entrepreneurial mindfulness is vital but insufficient in developing organizational resilience, and entrepreneurial resilience is necessary to transform the emotion and attention resources accumulated from mindfulness into entrepreneurs’ resilient behaviors which can foster organizational resilience. The observed partial mediation could also mean either a missing mediator, or both a direct and an indirect impact. A new door might be open for future researchers and policymakers to investigate what factors could fully mediate or moderate the paths.

Finally, our research extends UET in two aspects. Firstly, although the original UET proposed both psychological factors and observable factors as predictors for firm outcomes (Hambrick and Mason, 1984), the unobservable psychological ones are difficult to measure due to the reluctance of executives or entrepreneurs to participate in such survey (Hambrick and Mason, 1984). Therefore, even though UET has been recognized to play an important role in organization research, it is challenging to examine the black box of the mechanism between the entrepreneurs’ psychological characters and organizational outcomes (Hambrick, 2007; Nambisan and Baron, 2013; Chen et al., 2020; Liu et al., 2021). Our research adds to UET by bringing to bear first-hand survey data which measures the level of entrepreneurial mindfulness and resilience with widely used scales. Secondly, this paper enriches UET by solidifying the linkage between organizational resilience and entrepreneurship theory from combination of the psychological factors of entrepreneurs’ mindfulness and resilience, which has never been explored by previous studies.

### Managerial implications

Our results help to address the question: “How can Chinese SMEs develop their organizational resilience during the COVID-19 pandemic?.” In doing so, we suggest the need to pursue an integrative solution here. First, entrepreneurs of SMEs should act as effective crisis leaders who can keep a state of mindfulness when making decisions during such a crisis. They can participate in relevant mindfulness training programs and keep daily practices, through which they can concentrate on the current internal and external experiences, keep a positive attitude, fully mobilize resources and focus on solving problems. For example, effective mindfulness interventions for beginners such as Mindfulness-Based Stress Reduction (MBSR; Kabat-Zinn, 1990, 2013) and Mindfulness-based Cognitive Therapy (MBCT; Segal et al., 2002, 2013), can be hold at both the individual level and the enterprise level. In addition, meditations based on Buddhism or Taoism can also be effective forms of mindfulness practice. Both self-regulated and community-regulated ways are recommended to start and keep the practice. The effectiveness of mindfulness training or practice would be strengthened if it is accompanied with the emphasis on psychological resilience. Entrepreneurs must be fully aware of the importance of psychological resilience to the survival, success, and long-term development of enterprises. In doing so, entrepreneurs should lay emphasis on transforming the internal resources (attention and emotion) accumulated by mindfulness into external resilient actions against adversity. At the same time, supportive organizational environments should be nurtured to stimulate employee-level resilience across the organization and to promote the transmission of the resilient culture. For example, a flat organizational structure with less hierarchy is recommended. As a result, collective resilient behaviors will be aggregated into organizational resilient behaviors with a view of contributing to superior long-term performance.

In addition, we appeal for the active involvement of the government to offer initiative frameworks that can guide, direct, enable, and support SMEs to develop their capacity for resilience. In addition to making favorable regulations and policies such as tax and rent deductions, the government can also take measures to provide entrepreneurs of SMEs with relevant psychological and emotional supporting resources. For example, relevant guidance on entrepreneurial mindfulness and resilience can be conveyed through the official media. Non-profit projects for matching training and consulting agencies with SMEs can be sponsored and necessary subsidies should be offered.

Our findings can provide insights into how SMEs can promote organizational resilience in face of future crises and even the post-COVID period, which can also convey important implications for SMEs in other countries. It is enlightening in the practice of entrepreneurship cultivation and top executives’ selection in SEMs that individual mindfulness and resilience can be important characters that deserve the attention of human resource specialists. Moreover, the underlying mechanism revealed by our model can also shed light on the leadership cultivation and selection in large enterprises.

### Limitations and future research

Despite its strengths, our study has several limitations, some of which would provide directions for future research.

First, although common method bias is not a worrying issue in our data since we have followed specific and recommended methodological procedures (Podsakoff et al., 2003), future research should collect data from multiple respondents rather than only from entrepreneurs. Second, the sample only represents the Chinese target population, and thus the generalization of findings is limited because of the unique institutional, political and economic environments. Future research should address this limitation by conducting studies cross-nationally and including control variables that consider political, economic, and socio-cultural factors. Furthermore, this study did not consider the boundary conditions which could possibly create moderating effects in the proposed model. Future research should consider possible contextual or individual factors as moderators. Finally, as with early work, this study adopted only the subjectively self-reported measure of organizational resilience. Future research should adopt both subjective and objective measures of organizational resilience to replicate and extend the findings of this study.

## Conclusion

Based on UET and personality psychology, this study examines, in the context of Chinese SMEs during the COVID-19 pandemic, the influence of entrepreneurial mindfulness and entrepreneurial resilience on organizational resilience and the mediating role of entrepreneurial resilience between entrepreneurial mindfulness and organizational resilience. Firstly, this study is among the first to empirically explore why, how and when entrepreneurial mindfulness exerts positive effects on organizational resilience. By doing so, we extend the mindfulness theory at the individual level to the population of entrepreneurs and provide insights into the effect mechanism through which entrepreneurial mindfulness influences organizational resilience at the firm level. Secondly, this study enriches the body of research on organizational resilience by considering the joint influence of entrepreneurial mindfulness and entrepreneurial resilience. Thirdly, it extends UET by opening the back box of the mechanism between the entrepreneurs’ psychological characters and organizational resilience in the context of Chinese SMEs during the COVID-19 pandemic. Our findings can provide insights into how SMEs can promote organizational resilience in face of crises. These findings can convey important implications for SMEs in other countries with similar nature.

## Data availability statement

The dataset supporting the conclusions of this article is not publicly available. The dataset will be made available from the corresponding author on reasonable request.

## Ethics statement

Ethical review and approval were not required for the study on human participants in accordance with the local legislation and institutional requirements. Written informed consent for participation was not required for this study in accordance with the national legislation and the institutional requirements.

## Author contributions

XL: idea construction, writing, data analysis, and initial and final draft. XW: data collection. QW: funding and supervising. ZZ: editing and data analysis. All authors contributed to the article and approved the submitted version.

## Funding

The publication of this study was supported by the Research Project of Tianjin Education Commission for the project entitled “Retailer purchasing decision-making under the interactive effect of supply interruption risk and strategic customer behavior,” with the grant no. 2017SK071.

## Conflict of interest

The authors declare that the research was conducted in the absence of any commercial or financial relationships that could be construed as a potential conflict of interest.

## Publisher’s note

All claims expressed in this article are solely those of the authors and do not necessarily represent those of their affiliated organizations, or those of the publisher, the editors and the reviewers. Any product that may be evaluated in this article, or claim that may be made by its manufacturer, is not guaranteed or endorsed by the publisher.
