# Supplementary material for: Entrepreneurial mindfulness and organizational resilience of Chinese SMEs during the COVID-19 pandemic: The role of entrepreneurial resilience
Source: Front Psychol. 2022 Oct 6;13:992161. doi: 10.3389/fpsyg.2022.992161 (PMC9583948; doi:10.3389/fpsyg.2022.992161)
Supplement: Supplementary file 1 [file Table_1.DOCX]

| **Key clusters** | **Related researches** |
| --- | --- |
| **Individual level factors**  entrepreneurial characteristics (enterprise’s owner background, human capital , entrepreneurial orientation, entrepreneurial resilience, entrepreneurship, and social capital), employee life satisfaction, employee resilience, psychological capital, coping mechanisms | (Prayag et al., 2020; Branicki et al., 2017; Prayag et al., 2020; Herbane 2019; Wedawatta and Ingirige, 2016; Sun, 2011; Steiner and Cleary, 2014; Sköld and Tillmar, 2015; Richtnér and Löfsten, 2014; Pettit et al., 2013; Abylaev et al., 2014; Gunasekaran et al., 2011; Dumitraşcu and Dumitraşcu, 2016; Dillard et al., 2010; Demmer et al., 2011; Dahlberg and Guay, 2015; Crick and Crick, 2016; Chiesi, 2014; Bullough and Renko, 2013; Branzei and Abdelnour, 2010; Biggs et al., 2012b; Biggs et al., 2012a; Biggs, 2011; Biggs et al., 2015; Bernard and Barbosa, 2016; Ates and Bititci, 2011; Alonso and Bressan, 2015; Adnan et al., 2016; Acquaah et al., 2011; Alberti et al., 2018; Danes et al., 2009; Hedner et al., 2011; Littlewood and Holt, 2018; Mzid et al., 2018; Tengblad and Oudhuis, 2018; Tengblad, 2018; Torres et al., 2018) |
| **Group level factors**  collective mindfulness, collective rumination, group information processing | (Wang, 2021; Knipfer and Kump, 2022) |
| **Organizational level factors**  organizational ambidexterity, strategic consistency, social capital, dynamic capabilities, resourcefulness (material resources, financial resources, social resources, network resources, intangible resources), competitiveness (flexibility, redundancy of resources, robustness, networking), learning and culture, resource-based management initiative, endowment, business ethic, altruism, loss aversion, herd behavior, digitalization, and intellectual capital | (Iborra et al., 2020; Iborra et al., 2022; Ozanne et al., 2022; Huang et al., 2022; Syed et al., 2020; Winnard et al., 2014; Wedawatta and Ingirige, 2016; Tognazzo et al., 2016; Thomas et al., 2015; Sun, 2011; Sullivan-Taylor and Branicki, 2011; Smallbone et al., 2012; Sabatino, 2016; Abylaev et al., 2014; Ortiz-de-Mandojana and Bansal, 2016; Menéndez Blanco and Montes Botella, 2016; Lengnick-Hall et al., 2011; Lampel et al., 2014; Demmer et al., 2011; Crick and Crick, 2016; Conz et al., 2017; Carvalho et al., 2016; Biggs et al., 2012b; Biggs et al., 2012a; Biggs et al., 2015; Bernard and Barbosa, 2016; Akgün and Keskin, 2014; Acquaah et al., 2011; Alberti et al., 2018; Ali et al., 2017; Danes et al., 2009; Doeksen and Symes, 2015; Duchek, 2014; Hedner et al., 2011; Littlewood and Holt, 2018; Mzid et al., 2018; Tajuddin et al., 2017; Tengblad and Oudhuis, 2018; Tengblad, 2018) |
| **External business environment**  socio-cultures, institutions, macro-economic conditions, location, infrastructures, and environmental dynamism | (Do et al., 2022; Steiner and Cleary, 2014; Smallbone et al., 2012; Dillard et al., 2010; Branzei and Abdelnour, 2010; Biggs et al., 2012b; Biggs et al., 2012a; Biggs, 2011; Biggs et al., 2015; Alonso and Bressan, 2015; Akgün and Keskin, 2014; Adnan et al., 2016; Alberti et al., 2018; Ali et al., 2017) |

**Appendix**

**Table A1 Summary of key factors that influence organizational resilience of SMEs**

References in Table A1

Abylaev, M., Pal, R., and Torstensson, H. (2014). Resilience challenges for textile enterprises in a transitional economy and regional trade perspective-a study of Kyrgyz conditions. *International Journal of Supply Chain and Operations Resilience,* 1(1), 54-75. doi: 10.1504/IJSCOR.2014.065459

Acquaah, M.-G., KwasiJayaram, J., and Jayaram, J. (2011). Resilience in family and nonfamily firms: An examination of the relationships between manufacturing strategy, competitive strategy and firm performance. *International Journal of Production Research,* 49(18), 5527-5544. doi: 10.1080/00207543.2011. 563834

Adnan, A. H. M., Jaafar, R. E., Nasir, Z. A., and Mohtar, N. M. (2016). Just sisters doing business between us’: Gender, social entrepreneurship and entrepreneurial resilience in rural Malaysia. *International Journal of Entrepreneurship and Small Business,* 27(2/3), 273-288. doi: 10.1504/IJESB.2016.073992

Akgün, A. E., and Keskin, H. (2014). Organisational resilience capacity and firm product innovativeness and performance. *International Journal of Production Research,* 52(23), 6918-6937. doi: 10. 1080/00207543.2014.910624

Alberti, F. G., Ferrario, S., and Pizzurno, E. (2018). Resilience: Resources and strategies of SMEs in a new theoretical framework. *International Journal of Learning and Intellectual Capital,* 15(2), 165-188. doi: 10.1504/IJLIC.2018.091969

Ali, I., Nagalingam, S., and Gurd, B. (2017). Building resilience in SMEs of perishable product supply chains: Enablers, barriers and risks. *Production Planning & Control, 28(15),* 1236-1250. doi: 0.1080/ 09537287.2017.1362487

Alonso, A. D., and Bressan, A. (2015). Resilience in the context of Italian micro and small wineries: An empirical study. *International Journal of Wine Business Research,* 24(1), 40-60.

Ates, A., and Bititci, U. (2011). Change process: A key enabler for building resilient SMEs. *International Journal of Production Research,* 49(18), 5601-5618. doi: 10.1080/00207543.2011.563825

Bernard, M. J., and Barbosa, S. D. (2016). Resilience and entrepreneurship: A dynamic and biographical approach to the entrepreneurial act. *In Management (France).*

Biggs, D., (2011). Understanding resilience in a vulnerable industry: The case of reef Tourism in Australia. *In Ecology and Society,* 16(1).

Biggs, D., Ban, N., and C.Hall, C. M. (2012a). Lifestyle values, resilience, and nature-based tourism’s contribution to conservation on Australia’s Great Barrier Reef. *Environmental Conservation,* 39(4), 370-379. doi: 10.1017/S0376892912000239

Biggs, D., Hall, C. M., and Stoeckl, N. (2012b). ‘The resilience of formal and informal tourism enterprises to disasters: Reef tourism in Phuket, Thailand. *Journal of Sustainable Tourism,* 20(5), 645-665. doi: 10.1080/09669582.2011.630080

Biggs,, Hicks, C. C., Cinner, J. E., Hall, C. M., and Biggs, D. (2015). Marine tourism in the face of global change: The resilience of enterprises to crises in Thailand and Australia. *Ocean and Coastal Management,* 105, 65-74. doi: 10.1016/j.ocecoaman.2014.12. 019

Branicki, L. J., Sullivan-Taylor, B., and Livschitz, S. R. (2017). How entrepreneurial resilience generates resilient SMEs. *International Journal of Entrepreneurial Behavior & Research,* doi: 10.1108/IJEBR-11-2016-0396

Branzei, O., and Abdelnour, S. (2010). Another day, another dollar: Enterprise resilience under terrorism in developing countries. *Journal of International Business Studies,* 41(5), 804-825. doi: 10.1057/jibs.2010.6

Bullough, A., and Renko, M. (2013). Entrepreneurial resilience during challenging times. *Business Horizons.*

Carvalho, A. O., Ribeiro, I., Cirani, C. B. S., and Cintra, R. F. (2016). Organizational resilience: A comparative study between innovative and non-innovative companies based on the financial performance analysis. *International Journal of Innovation,* 4(1), 58. doi.org/10.5585/iji.v4i1.73

Chiesi, A. M. (2014). Interpersonal networking and business resilience: How immigrants in small business face the crisis in Italy. *European Sociological Review,* 30(4), 457-469. doi: 10.1093/esr/jcu052

Conz, E., Denicolai, S., and Zucchella, A. (2017). The resilience strategies of SMEs in mature clusters. *Journal of Enterprising Communities: People and Places in the Global Economy,* 11(1), 186-210. doi: 10. 1108/JEC-02-2015-0015

Crick, J. M., and Crick, D. (2016). Developing entrepreneurial resilience in the UK tourism sector. *In Strategic change.*

Dahlberg, R., and Guay, F. (2015). ‘Creating resilient SMEs: Is business continuity management the answer? *WIT Transactions on the Built Environment,* 168, 975-984.

Danes, S. M., Lee, J., Amarapurkar, S., Stafford, K., Haynes, G., and Brewton, K. E. (2009). Determinants of family business resilience after a natural disaster by gender of business owner. *Journal of Developmental Entrepreneurship,* 14(4), 333-354. doi: 10. 1142/S1084946709001351

Demmer, W. A., Vickery, S. K., and Calantone, R. (2011). Engendering resilience in small-and medium-sized enterprises (SMEs): A case study of Demmer Corporation. *International Journal of Production Research,* 49(18), 5395-5413. doi: 10. 1080/00207543.2011.563903

Dillard, J., Pullman, M. E., Stubblefield Loucks, E., Martens, M. L., Cho, C. H. et al. (2010). Engaging small-and medium-sized businesses in sustainability. *Sustainability Accounting, Management and Policy Journal,* 1(2), 178-200. doi: 10.1108/20408021011089239

Do, H., Budhwar, P., Shipton, H., Nguyen, H. D., and Nguyen, B. (2022). Building organizational resilience, innovation through resource-based management initiatives, organizational learning and environmental dynamism. *Journal of Business Research,* 141, 808-821. doi: 10.1016/j.jbusres.2021.11.090

Doeksen, A., and Symes, D. (2015). Business strategies for resilience: The case of Z eeland’s Oyster industry. *Sociologia Ruralis,* 55(3), 325-342. doi: 10. 1111/soru.12099

Duchek, S. (2014). ‘Growth in the face of crisis: The role of organizational resilience capabilities.’ Paper presented at Academy of Management Proceedings, 2014(1), 13487-10510. *Academy of Management,* doi: 10.5465/ambpp.2014.225

Dumitraşcu, V., and Dumitraşcu, R. A. (2016). The use of fuzzy sets and elements of the information theory for assessing the resilience level of business organizations. *In Quality - Access to success,* 17, 69-85.

Gunasekaran, A., Rai, B. K., and Griffin, M. (2011). Resilience and competitiveness of small and medium size enterprises: An empirical research. *International Journal of Production Research,* 49(18), 5489-5509. doi: 10.1080/00207543.2011.563831

Hedner, T., Abouzeedan, A., and Klofsten, M. (2011). *Entrepreneurial resilience.*

Herbane, B. (2019). Rethinking organizational resilience and strategic renewal in SMEs. *Entrepreneurship & Regional Development,* 31(5-6), 476-495. doi: 10.1080/08985626.2018.1541594

Huang, X., Chau, K. Y., Tang, Y. M., and Iqbal, W. (2022). Business ethics and irrationality in SME during COVID-19: does it impact on sustainable business resilience?. *Frontiers in Environmental Science,* 10. doi: 10.3389/fenvs.2022.870476

Iborra, M., Safón, V., and Dolz, C. (2020). What explains the resilience of SMEs? Ambidexterity capability and strategic consistency. *Long Range Planning,* 53(6), 101947. doi: 10.1016/j.lrp.2019.101947

Iborra, M., Safón, V., and Dolz, C. (2022). Does ambidexterity consistency benefit small and medium-sized enterprises’ resilience?. *Journal of Small Business Management,* 1-44. doi: 10.1080/00472778.2021.2014508

Knipfer, K., and Kump, B. (2022). Collective rumination: When “problem talk” impairs organizational resilience. *Applied Psychology,* 71(1), 154-173. doi: 10.1111/apps.12315

Lampel, J., Bhalla, A., and Jha, P. P. (2014). Does governance confer organisational resilience? Evidence from UK employee owned businesses. *European Management Journal,* 32(1), 66-72. doi: 10.1016/j.emj. 2013.06.009

Lengnick-Hall, C. A., Beck, T. E., and Lengnick-Hall, M. L. (2011). Developing a capacity for organizational resilience through strategic human resource management. *Human Resource Management Review,* 21(3), 243-255. doi: 10.1016/j.hrmr.2010.07.001

Littlewood, D., and Holt, D. (2018). Social enterprise resilience in sub-Saharan Africa. *Business Strategy & Development,* 1 (1), 53-63. doi: 10.1002/bsd2.11

Menéndez Blanco, J. M., and Montes Botella, J. L. (2016). What contributes to adaptive company resilience? A conceptual and practical approach. *In Development and Learning in Organizations,* 30(4), 17- 20. doi: 10.1108/DLO-10-2015-0080

Mzid, I., Khachlouf, N., and Soparnot, R. (2018). How does family capital influence the resilience of family firms? *Journal of International Entrepreneurship,* 17(2), 249-277. doi: 10.1007/s10843-018-0226-7

Mzid, I., Khachlouf, N., and Soparnot, R. (2018). How does family capital influence the resilience of family firms? *Journal of International Entrepreneurship,* 17(2), 249-277. doi: 10.1007/s10843-018-0226-7

Ortiz-de-Mandojana, N., and Bansal, P. (2016). The long-term benefits of organizational resilience through sustainable business practices. *Strategic Management Journal,* 37(8), 1615-1631. doi: 10.1002/smj.2410

Ozanne, L. K., Chowdhury, M., Prayag, G., and Mollenkopf, D. A. (2022). SMEs navigating COVID-19: The influence of social capital and dynamic capabilities on organizational resilience. *Industrial Marketing Management,* 104, 116-135. doi: 10.1016/j.indmarman.2022.04.009

Pettit, T. J., Croxton, K. L., and Fiksel, J. (2013). Ensuring supply chain resilience: Development and implementation of an assessment tool. *Journal of Business Logistics,* 34(1), 46-76. doi: 10.1111/jbl. 12009

Prayag, G., Ozanne, L. K., and de Vries, H. (2020). Psychological capital, coping mechanisms and organizational resilience: Insights from the 2016 Kaikoura earthquake, New Zealand. *Tourism Management Perspectives,* 34, 100637. doi: 10.1016/j.tmp.2020.100637

Prayag, G., Spector, S., Orchiston, C., and Chowdhury, M. (2020). Psychological resilience, organizational resilience and life satisfaction in tourism firms: Insights from the Canterbury earthquakes. *Current Issues in Tourism,* 23(10), 1216-1233. doi: 10.1080/13683500.2019.1607832

Richtnér, A., and Löfsten, H. (2014). Managing in turbulence: How the capacity for resilience influences creativity. *In R and D management,* 44(2), 137-151. doi: 10.1111/radm.12050

Sabatino, M. (2016). Economic crisis and resilience: Resilient capacity and competitiveness of the enterprises. *Journal of Business Research, 69(5),* 1924-1927. doi: 10.1016/j.jbusres.2015. 10.081

Sköld, B., and Tillmar, M. (2015). Resilient gender order in entrepreneurship: The case of Swedish welfare industries. *International Journal of Gender and Entrepreneurship,* 7(1), 2-26. doi: 10.1108/ IJGE-09-2013-0057

Smallbone, D., Deakins, D., Battisti, M., and Kitching, J. (2012). Small business responses to a major economic downturn: Empirical perspectives from New Zealand and the United Kingdom. *International Small Business Journal: Researching Entrepreneurship,* 30 (7), 754-777. doi: 10.1177/ 0266242612448077

Steiner, A. A., and Cleary, J. (2014). What are the features of resilient businesses? Exploring the perception of rural entrepreneurs. *Journal of Rural and Community Development,* 9(3), 1-20.

Sullivan-Taylor, B., and Branicki, L. (2011). Creating resilient SMEs: Why one size might not fit all. *International Journal of Production Research,* 49(18), 5565-5579. doi: 10.1080/00207543.2011.563837

Sun, J. B., Buys,, Nicholas, X., McAuley, A., and Buys, N. (2011). Using the concept of resilience to explain entrepreneurial success in China. *International Journal of Management and Enterprise Development,* 11(2/3/4), 182-202. doi: 10.1504/IJMED. 2011.044637

Syed, H. A., Schorch, M., Hassan, S. S., Skudelny, S., Grinko, M., et al. (2020). From technology adoption to organizational resilience: A current research perspective. doi: 10.25819/ubsi/2778

Tajuddin, R. M., Hashim, S. F., and Zainol, A. S. (2017). The role of brand identity in creating resilient small enterprises (SMEs) in fashion industry. *International Journal of Supply Chain Management,* 6(2), 140-146.

Tengblad, S. (2018). A resource-based model of organizational resilience. *In The Resilience Framework,* 39-54. doi: 10.1007/978-981- 10-5314-6_3

Tengblad, S., and Oudhuis, M. (2018). Organization resilience: What makes companies and organizations sustainable? *In The Resilience Framework,* 3-17. doi: 10.1007/978-981-10-5314- 6_1.

Thomas, A., Pham, D. T., Francis, M., and Fisher, R. (2015). Creating resilient and sustainable manufacturing businesses-a conceptual fitness model. *International Journal of Production Research,* 53 (13), 3934-3946. doi: 10.1080/00207543. 2014.975850

Tognazzo, A., Gubitta, P., and Favaron, S. D. (2016). Does slack always affect resilience? A study of quasi-medium-sized Italian firms. *Entrepreneurship & Regional Development,* 28(9-10), 768-790. doi: 10.1080/08985626.2016.1250820

Torres, A. P., Marshall, M. I., and Sydnor, S. (2018). Does social capital pay off? The case of small business resilience after Hurricane Katrina. *Journal of Contingencies and Crisis Management,* 27(2), 168-181. doi: 10.1111/1468-5973.12248

Wang, L., Müller, R., Zhu, F., and Yang, X. (2021). Collective Mindfulness: The Key to Organizational Resilience in Megaprojects. *Project Management Journal,* 52(6), 592-606. doi: 10.1177/87569728211044908

Wedawatta, G., and Ingirige, B. (2016). A conceptual framework for understanding resilience of construction SMEs to extreme weather events. *Built Environment Project and Asset Management,* 6(4), 428-443. doi: 10.1108/BEPAM-06-2015-0023

Winnard, J., Adcroft, A., Lee, J., and Skipp, D. (2014). Surviving or flourishing? Integrating business resilience and sustainability. *Journal of Strategy and Management,* 7(3), 303-315. doi: 10.1108/ JSMA-11-2012-0059
